# Cardiac rehabilitation to improve health-related quality of life following trans-catheter aortic valve implantation: a randomised controlled feasibility study: RECOVER–TAVI Pilot, ORCA 4, For the Optimal Restoration of Cardiac Activity Group

**DOI:** 10.1186/s40814-018-0363-8

**Published:** 2018-12-13

**Authors:** Paula Rogers, Sayed Al-Aidrous, Winston Banya, Shelley Rahman Haley, Tarun Mittal, Tito Kabir, Vasileois Panoulas, Shahzad Raja, Sunil Bhudia, Heather Probert, Claire Prendergast, Mark S. Spence, Simon Davies, Neil Moat, Rod S. Taylor, Miles Dalby

**Affiliations:** 1 Royal Brompton & Harefield NHS Foundation Trust London, Imperial College, London, UK; 2 Royal Victoria Hospital, Belfast, UK; 3 Institute of Health Research, University of Exeter Medical School, Exeter & The School of Medicine, Dentistry & Nursing, University of Glasgow, Glasgow, UK

**Keywords:** Cardiac rehabilitation, Transcatheter aortic valve implantation, Randomised controlled trial, Pilot study

## Abstract

**Objectives:**

Transcatheter aortic valve implantation (TAVI) is often undertaken in the oldest frailest cohort of patients undergoing cardiac interventions. We plan to investigate the potential benefit of cardiac rehabilitation (CR) in this vulnerable population.

**Design:**

We undertook a pilot randomised trial of CR following TAVI to inform the feasibility and design of a future randomised clinical trial (RCT).

**Participants:**

We screened patients undergoing TAVI at a single institution between June 2016 and February 2017.

**Interventions:**

Participants were randomised post-TAVI to standard of care (control group) or standard of care plus exercise-based CR (intervention group).

**Outcomes:**

We assessed recruitment and attrition rates, uptake of CR, and explored changes in 6-min walk test, Nottingham Activities of Daily Living, Fried and Edmonton Frailty scores and Hospital Anxiety and Depression Score, from baseline (30 days post TAVI) to 3 and 6 months post randomisation. We also undertook a parallel study to assess the use of the Kansas City Cardiomyopathy Questionnaire (KCCQ) in the post-TAVI population.

**Results:**

Of 82 patients screened, 52 met the inclusion criteria and 27 were recruited (3 patients/month). In the intervention group, 10/13 (77%) completed the prescribed course of 6 sessions of CR (mean number of sessions attended 7.5, SD 4.25) over 6 weeks. At 6 months, all participants were retained for follow-up. There was apparent improvement in outcome scores at 3 and 6 months in control and CR groups. There were no recorded adverse events associated with the intervention of CR. The KCCQ was well accepted in 38 post-TAVI patients: mean summary score 72.6 (SD 22.6).

**Conclusions:**

We have demonstrated the feasibility of recruiting post-TAVI patients into a randomised trial of CR. We will use the findings of this pilot trial to design a fully powered multicentre RCT to inform the provision of CR and support guideline development to optimise health-related quality of life outcomes in this vulnerable population. Retrospectively registered 3rd October 2016 clinicaltrials.gov NCT02921880.

**Trial registration:**

Clinicaltrials.Gov identifier NCT02921880

**Electronic supplementary material:**

The online version of this article (10.1186/s40814-018-0363-8) contains supplementary material, which is available to authorized users.

## Introduction

The prevalence of aortic stenosis (AS) is rising in an ageing population and carries significant risk [1, 2]. If left untreated, symptomatic severe aortic stenosis can have a mortality of 75% at 3.5 years with up to 50% of dying suddenly [3].

Transcatheter aortic valve implantation (TAVI) has been shown to be superior to medical management and at least equivalent to surgical replacement in high and intermediate risk patients for short and intermediate term clinical outcomes [4]. As a consequence, TAVI has caused a paradigm shift by providing a percutaneous alternative to surgical aortic valve replacement and over the past 15 years over 100,000 TAVI procedures have been undertaken with recent-meta analysis reporting a mean patient age of 81.5 years [5].

The World Health Organization defines health as not merely the absence of disease but rather a more complex interplay between social and medical factors resulting in a holistic sense of wellbeing [6]. This interaction is of greater consequence in the elderly whose frailty, multiple co-morbidities, advanced age and reduced reserve result increased procedural morbidity and mortality [7, 8]. Whilst pivotal randomised trials focus on five key outcome measures of mortality, stroke, paravalvar leak, pacemaker requirement and vascular access complications, these are not the sole metrics of clinical success: Hospital readmission is common occurring in up to half of patients at 1 year with a high prevalence of heart failure and significant morbidity [9]. Furthermore, heart failure patients regard good levels of functional outcome and health-related quality of life (HRQoL) equally or more important than improvements in mortality [10]. Large randomised studies have addressed disease specific and generic measures of HRQoL but these have been limited to the impact of the TAVI intervention itself rather than optimising the subsequent outcome [11]. Frailty is a particularly important syndrome of multiple impairments in patients with cardiovascular disease [12] and whilst difficult to quantify is associated with increased vulnerability to stressors [13].

Cardiac rehabilitation (CR) is a multi-faceted intervention that not only improves quality of life and reduces depression, but has also been shown to reduce hospital admissions and disease-specific mortality in post-myocardial infarction, revascularisation and heart failure populations [14–16]. As a consequence, CR is mandated in national and international guidelines for these indications [17–20]. Although some small studies have been reported [21, 22], CR has not yet been investigated in in post TAVI patients in a randomised controlled trial (RCT) powered on HRQoL or clinical events. CR’s focus on education, lifestyle intervention and exercise renders it particularly important in frailer higher risk individuals who undergo TAVI. In addition, the elderly are often under-represented in clinical trials with trial design, logistical and financial factors being cited as obstacles to recruitment [23].

We therefore undertook a pilot RCT to inform the feasibility and design of a future fully outcome powered multicentre randomised trial of CR after TAVI. Specific study objectives were to (1) assess recruitment rates; (2) assess acceptability and uptake of CR and quantify the delivery of CR in this population; (3) assess attrition rates; (4) explore the impact of CR on functional, independence, frailty and emotional outcomes 30 days post TAVI (baseline) and post-CR at 3 and 6 months; and (5) assess suitability of the use of the Kansas City Cardiomyopathy Questionnaire (KCCQ) in a separate post TAVI cohort.

## Methods

This study is reported in accordance with the CONSORT extension for pilot and feasibility trials [24] (Additional file 1).

### Trial design

RECOVER-TAVI is a single-centre pilot RCT in patients who have undergone TAVI. Patients were randomly allocated 4 weeks after their TAVI procedure in a 1:1 ratio with allocation administered by an investigator independent of the study, stratified by age and gender to either standard of care (SOC; control group) or CR plus SOC (intervention group). Outcomes were measured 30 days post TAVI (baseline), and at 3 and 6 months post randomisation by an assessor blinded to group allocation.

### Participants

All patients scheduled for TAVI at our centre between June 2016 and March 2017 were screened for trial inclusion. Patients were included if they fulfilled the following: Severe symptomatic aortic stenosis accepted for TAVI in our institutional Multidisciplinary Team Meeting, age ≥ 75 years, able to give written informed consent, and in the Investigator’s opinion, able to comply with all study requirements. Study exclusions were CR deemed inappropriate due to co-morbidity or frailty, life expectancy less than 1 year due to co-morbidity, previous AVR or TAVI, or predominant aortic regurgitation.

### Ethics approval and consent to participate

Written informed consent was given prior to the TAVI procedure (rather than during post procedure recovery) (Additional file 2).

### Control and intervention groups

Patients randomised to the control group received SOC according to our institutional protocols. Patients randomised to the intervention group underwent a comprehensive biopsychosocial assessment with a member of the exercise team, initiated 1-month post procedure and comprised of once weekly sessions for 60–90 min for six sessions. An individualised programme was prescribed for each patient based on information gained from their functional capacity test and discussion around their specific goals. Exercise was prescribed following the Frequency, Intensity, Time and Type (FITT) principle and a moderate intensity interval approach was used based on the Association of Chartered Physiotherapists in Cardiac Rehabilitation (ACPICR) Standards [25]. To monitor exercise response, ambulatory 3 lead ECG recordings were recorded (C.Net 5000, Cardionetics, UK), heart rate (HR) monitors (Polar Watch, Polar T31 FS2, Polar Electro, Finland) and the Borg rating of perceived exertion scale were used (ACPICR). Exercise prescription consisted of graduated cardiovascular training and resistance training (both upper and lower body) using cardiovascular exercise machines (treadmill and bike) as well as functional exercise such as ‘sit to stand’. After each exercise session, each individual’s prescription was reviewed and altered appropriately for the subsequent session. The intensity of the exercise was progressively increased based on the self-reported BORG intensity.

Patients were offered further sessions if able to attend, in line with our institutional programme and the British Association for Cardiovascular Prevention and Rehabilitation (BACPR) recommendations [26, 27]. Prior to 1-month post-TAVI, patients did not undergo formal rehabilitation though were given general advice regarding early mobilisation as per usual care.

Both control and intervention groups received routine medical care which included an outpatient clinic follow-up appointment, appropriate drug therapy and concomitant medical management of co-morbidities according to local practice.

### Outcomes

Baseline characteristics were collected at randomisation (30 days post TAVI) and study outcomes measured at baseline (pre-randomisation), and 3 months and 6 months post-randomisation. Feasibility outcomes of this study were: recruitment rate (i.e. average number of patients/month willing to give informed consent and be randomised into a trial of CR post TAVI); acceptability and uptake of CR and quantification the delivery of CR; and attrition rates (i.e. proportion of patients who provided outcomes at 3- and 6-months follow-up). We collected the following patient-related outcomes: 6-minute walk test (6MWT) [28], Nottingham Activities of Daily Living (ADL, scale of 0 for least activity to 22 for most activity) [29], FRIED Frailty score (0 = not frail, 1–2 = pre-frail, 3 = frail) [30], Edmonton Frailty Score (9 domains, scale of 0 for non-frail to 17 for severely frail) [31] and Hospital Anxiety and Depression Scores (HADS, 0–7 normal, 8–10 borderline, 11–21 abnormal) Score [32].

Following the start of the pilot study, due to the difficulty in measuring the study outcome measures and considering the primary objective of informing the design of a subsequent clinical outcome powered study we decided to incorporate a disease-specific HRQoL parallel observational sub-study to assess the acceptability and applicability of the KCCQ in a cohort of post-TAVI patients [33].

### Blinding

Given the nature of the intervention in this study, neither participants nor clinicians were blinded to group allocation. Outcome assessors were however blinded to group.

### Statistical methods

Given the pilot design of this study we based the sample size on the feasibility objective of informing the planning of a definitive trial, rather than on formal power calculation to detect between-group difference for patient outcomes. Considering a likely ‘moderate’ effect size of the CR intervention we estimated that approximately 15 patients per group would be required to inform the design of a definitive trial [34]. Patient reported outcomes for both groups were reported descriptively according to intention-to-treat principle (i.e. according to initial random allocation) at all time points using the mean (standard deviation, SD) or median (range). Categorical data were presented as number (%). Between group differences at follow-up were estimated by the difference in means between the 2 groups and presented as means and 95% CIs. All data analyses were undertaken using version 14.1 of Stata software [35].

### Patient and public involvement

Patients and their carers were involved in the study design before the application for funding was submitted.

The lay summary was prepared and discussed with patients in the out-patient department and ward areas. Patients with and without aortic stenosis were approached to comment on the study design. Overall, the patients who were surveyed said that they would be happy to be approached to take part in the study. Some patients were happy that the choice to undertake cardiac rehabilitation would be re-affirmed at 4 weeks post implant, rather than in the immediate post-operative period. Some patients felt that the study offered a robust plan of follow-up in which patients would feel reassured. Travel expenses were commented on as being an issue for some people and it was explained that a budget would be secured to reimburse travel costs.

The patient feedback was incorporated into the Research Ethics Committee application and the overall study design. Patients were invited to receive a lay summary of the study findings at the end of the project.

## Results

**Fig. 1:**
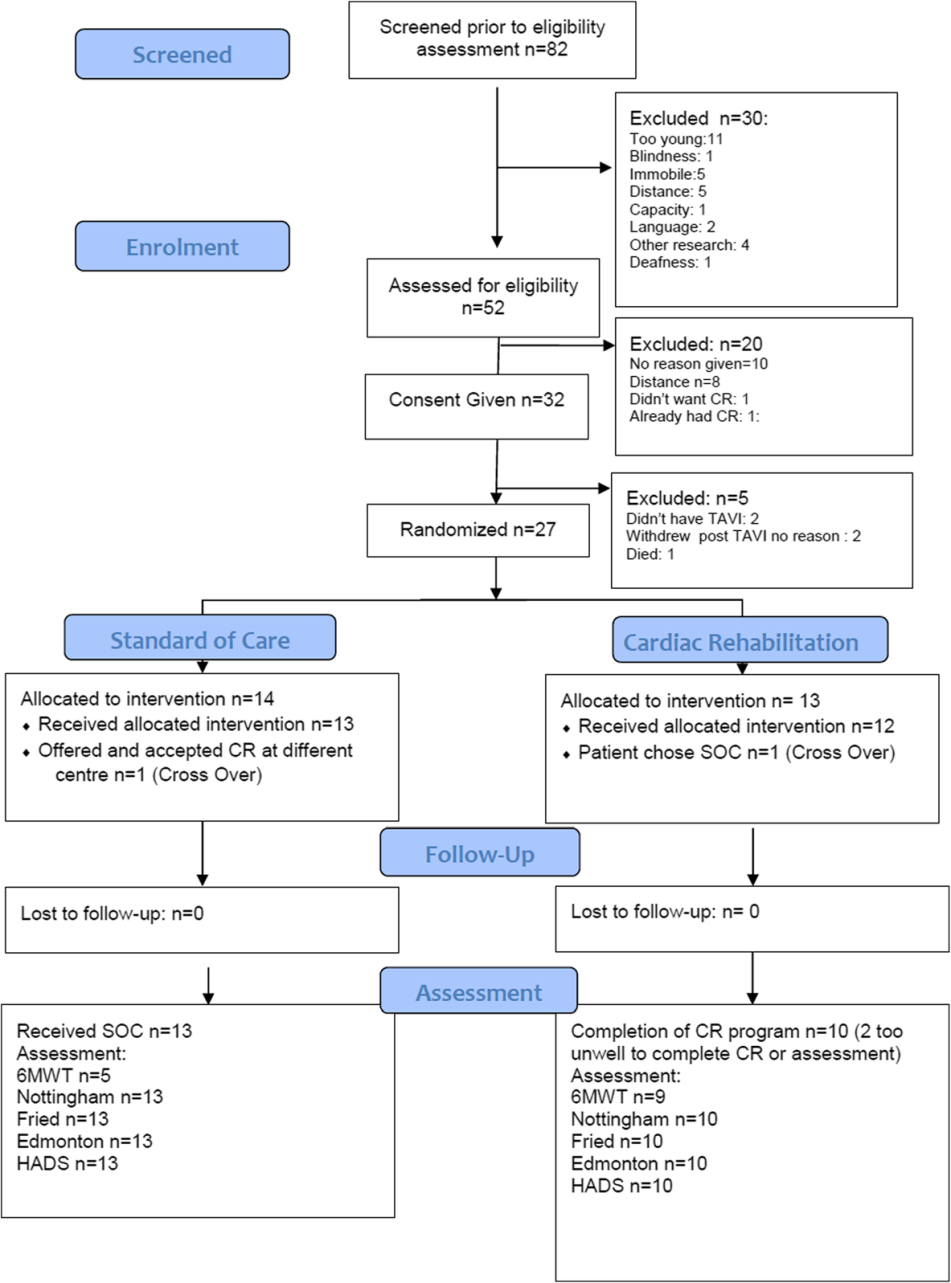
ᅟRECOVER-TAVI CONSORT flow diagram

A total of 82 patients scheduled for TAVI were screened of whom 52 were eligible for study recruitment. Of these, 20 declined; most frequently due to concerns about travel or they chose not to give a specific reason. Study recruitment was extended from 30 to 32 because two patients ultimately did not undergo TAVI for technical reasons. Three patients withdrew after TAVI; two for no given reason and one died. Therefore, 27 patients were randomised (33% of those screened). Following randomisation one participant in each group switched to the other arm through patient choice. Thirteen of the control group patients completed the study assessment. Ten of the 13 Intervention group completed the CR and assessment, three being too unwell to do so, and all patients were followed up. Ultimately therefore, 23 patients (28% of those screened) were enrolled in the study and completed the study interventions, with the exception that only 14 of the study patients underwent the 6MWT due to frailty (Fig. 1 and Table 1).

**Table 1:** Baseline characteristics for randomised pilot study

	All Participants (*n* = 27)	Control group (*n* = 14)	Intervention group (*n* = 13)
Male, *n* (%)	12 (44.4)	6 (42.9)	6 (46.2)
Age: mean (SD)	82.04 (4.8)	81.21 (3.6)	82.92 (6.0)
BMI: mean (SD)	27.70 (4.2)	28.13 (4.9)	27.24 (3.6)
Diabetes, *n* (%)	4 (14.8)	3 (21.4)	1 (7.7)
Smoking: never *n* (%)	13 (48.1)	7 (50.0)	6 (46.2)
Ex, *n* (%)	14 (51.9)	7 (50.0)	7 (53.8)
Creatinine: mean (SD)	95.3 (28.1)	90.0 (23.9)	101.0 (32.0)
Previous MI, *n* (%)	5 (18.5)	2 (14.3)	3 (23.1)
History of pulmonary disease, *n* (%)	7 (25.9)	4 (28.6)	3 (23.1)
Pre-operative arrhythmia, *n* (%)	15 (55.6)	7 (50.0)	8 (61.5)
Previous cardiac surgery, *n* (%)	7 (25.9)	3 (21.4)	4 (30.8)
Previous PCI, *n* (%)	11 (40.7)	5 (35.7)	6 (46.2)
Left ventricular ejection fraction, *n* (%)			
≥ 50%	21 (77.8)	12 (85.7)	9 (69.2)
30–49%	5 (18.5)	2 (14.3)	3 (23.1)
< 30%	1 (3.7)	0	1 (7.7)
6-min walk (metres)*	346.9(44.7)	401.0(41)	325.2(22.8)
FRIED scale 0 n (%)	3/25 (12.0)	2/12 (16.7)	1 (7.7)
1, *n* (%)	9/25 (36.0)	3/12 (25.0)	6 (46.2)
2, *n* (%)	8/25 (32.0)	5/12 (41.7)	3 (23.1)
3, *n* (%)	5/25 (20.0)	2/12 (16.7)	3 (23.1)
Nottingham EADL (0–22) *	15.7 (3.9)	14.8 (4.7)	16.5 (3.0)
Edmonton Frail Scale (0–17) *	5.17 (1.9)	5.25 (1.8)	5.08 (2.2)
HADS Anxiety (0–21) **	3.0 (2, 4)	3.0 (2, 5)	3.0 (2, 4)
HADS Depression (0–21) **	2.0 (1, 5)	2.0 (1, 4)	2.0 (2, 5)

*Mean (SD)

**Median (inter-quartile range)

### Baseline characteristics

The overall study mean age was 82 years and the majority of participants (55%) were male. There was some evidence of imbalance in baseline outcome scores between groups, with 6-min walk distance being greater and left ventricular ejection fraction being numerically greater in the control group than the intervention group (Table 1, Additional file 3) most likely due to the play of chance in small groups.

### Recruitment and attrition

**Fig. 2:**
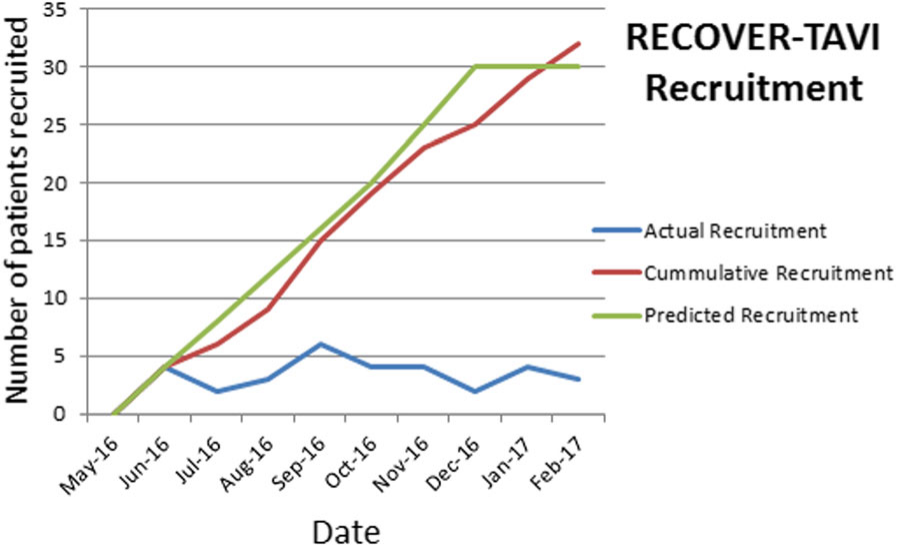
ᅟRECOVER TAVI recruitment timeline

- Thirty-two patients were recruited over 10 months indicating a recruitment rate of 3.2 patients per month (Fig. 2)
- There was 100% retention of participants in the study and no loss to follow up (Fig. 1).

### CR delivery and follow-up

Ten (77%) participants in the intervention group completed the prescribed course of 6 sessions. The average number of sessions completed was 7.5 (SD 4.25). Three participants completed more than the six prescribed sessions attending 15, 13 and 12 sessions respectively. We were able to retain all control and intervention participants over the 6-months of the study and no patients were lost to follow. One control participant died prior to the 6 month follow-up due to a non-cardiac cause. The logistical considerations and costs of the CR intervention were defined. There were no recorded adverse events associated with the intervention of CR.

### Functional, Independence, frailty and emotional outcomes

There were improvements at 3 and 6 months in 6-min walk test, Nottingham activities of daily living, FRIED Frailty Score, Edmonton frailty score and Hospital Anxiety and Depression score in both groups. There was no apparent difference between the control and intervention groups however at 3 or 6 months (Tables 2 and 3 Additional file 4).

**Table 2:** Outcomes at 3 months

	Control group *N* = 14	Intervention group *N* = 13	Treatment effect (95% CI)
6-min walk (metres) *	370.0 (33)	319.7 (24.5)	− 50.33 (− 171.1, 70.4)
FRIED Scale 0	1/14 (7.1%)	2/11 (18.2%)	
1	6/14 (42.9%)	5/11 (45.4%)	
2	6/14 (42.9%)	1/11 (9.1%)	
3	1/14 (7.1%)	3/11 (27.3%)	
Nottingham EADL (0–22)*	17.8 (1.8)	18.2 (2.5)	0.4 (−1.4, 2.1)
Edmonton Frail Scale (0–17)*	4.50 (2.0)	4.4 (1.7)	− 0.1 (−1.7, 1.4)
HADS Anxiety (0–21)**	2 (1, 5)	3 (2, 4.5)	0 (−3, 2)
HADS Depression (0–21)**	3.5 (1, 5)	3.5 (2, 6.5)	1 (−2, 3)

*Mean (SD)

**Median (inter-quartile range)

**Table 3:** Outcomes at 6 months

	Control group *N* = 14	Intervention group *N* = 13	Treatment effect (95% CI)
6-min walk (metres)*	385.5 (84.3)	292.9 (24.5)	−92.6 (− 206.7, 21.4)
FRIED Scale 0	4/12	2/13	
1	6/12	4/13	
2	2/12	3/13	
3	0	4/13	
Nottingham EADL (0–22) *	17.33 (2.9)	18.0 (2.3)	0.7 (−1.5, 2.8)
Edmonton Frail Scale (0–17) *	4.58 (1.9)	5.15 (2.4)	0.6 (−1.2, 2.3)
HADS Anxiety (0–21) **	3.0 (2, 5)	3 (2, 4)	0 (−2, 2)
HADS Depression (0–21) **	2 (1,45)	2 (2, 5)	1 (0, 3)

*Mean (SD)

**Median (Inter-quartile range)

### Adverse events

There were no recorded adverse events associated with the intervention group.

### TAVI KCCQ sub-study

Thirty-eight separate post-TAVI patients completed the KCCQ with a mean clinical summary score of 73.6 (SD 21.6) and mean overall summary score of 72.6 (SD 22.6) (Table 4, Additional file 5). The characteristics of participants in this sub-study were similar to those in the pilot study.

**Table 4:** Characteristics of *N* = 38 participants for the Kansas City Cardiomyopathy Questionnaire sub-study

Variable	
Female (%)	21 (55.3)
Age: mean (SD)	81.9 (7.3)
BMI: mean (SD)	26.8 (6.0)
Diabetes (%)	8(21.1)
Smoking (%): never	21 (55.3)
Ex	17 (44.7)
Creatinine: mean (SD)	90.9 (33.6)
Previous MI (%)	10 (26.3)
History of pulmonary disease	12 (31.6)
Pre-operative arrhythmia	17 (44.7)
Previous cardiac surgery	13 (34.2)
Previous PCI	9 (23.7)
Left Ventricular Ejection Fraction:	
≥ 50%	32 (84.2)
30–49%	5 (13.1)
< 30%	1 (2.6)
KCCQ overall summary: mean (SD)	72.6 (22.6)
KCCQ clinical summary: mean (SD)	73.6 (21.6)

## Discussion

We achieved the primary objective of this pilot study and demonstrated the feasibility and acceptability of recruitment and retention in an elderly post TAVI cohort of participants in the context of a randomised clinical trial of CR.

We also defined logistic considerations and costs around delivering CR for this post TAVI population in the context of a future randomised trial.

There were baseline imbalances in 6-min walk test, and left ventricular function between the groups which may have affected the outcome and the study was not powered to measure the efficacy of CR.

There were a number of key learning points which will inform the design of an outcome powered study:

The investigators, although experienced in clinical frailty assessment reported that questionnaires were not always easy to administer and quantifiable differences between the scores were not easy to measure, especially for frailty scores.

In the parallel KCCQ clinical quality HRQoL audit the average age and comorbidity profile was similar to the trial cohort and the Overall Summary and Clinical Summary KCCQ score and standard deviation were defined. In particular, the KCCQ level was similar to that reported in large clinical trials following TAVI and is likely to generalizable to a wider multicentre population for a subsequent trial.

Of note, the CR intervention was started at day 30 post-TAVI because of concerns about vascular access discomfort/complications. In the event, it was felt by the CR team that most patients could have started the CR earlier when potentially its impact would have been greater.

Anecdotally, the CR team were often asked about medical management such as clarifying drug regimens and where necessary, opportunistic scheduling of clinic appointments was facilitated potentially reducing readmission rates.

Our study has a number of limitations. There was a disparity in the baseline characteristics between the control and intervention groups. As it was a single-centre trial, our findings have limited generalisability—though we adopt similar treatment pathways and clinical protocols to those used widely in the UK including by groups who have indicated willingness to engage in a multicentre trial. Given the small sample size, the study was not powered to formally compare the changes in outcomes within or between groups. We could have studied more outcomes including the KCCQ and others in the randomised trial but administering the assessment tools is time consuming and patient fatigue becomes a significant problem reducing both quality of assessment and patient acceptance, thus we studied the KCCQ in a separate audit of similar patients.

In this pilot we elected first to assess the acceptability to this frail cohort of a relatively modest intensity and duration of CR. Although this was a pilot study not powered for HRQoL or frailty outcomes, the lack of any signal of difference between the CR and SOC groups might suggest that intensifying the CR intervention may be appropriate. In addition, the fact that the majority of patients completed the CR course and that some elected for further CR indicates that a more intensive and longer course may be acceptable to this cohort for a subsequent trial.

### Implications for planning a future trial

This pilot study has demonstrated the feasibility of performing a multicentre trial of SOC vs CR in a post TAVI population.

We were able to recruit 3 patients per month into the study indicating that over a two-year period we could anticipate recruiting 72 patients. The pilot and proposed outcome powered trial have been presented at a National Meeting and ten centres approached have indicated willingness to participate.

Specifically, CR was acceptable to this patient cohort with excellent participant retention. We are planning a multicentre outcome powered HRQoL study of SOC vs CR for patients who have received TAVI to inform clinical practice, optimise patient outcomes and support guideline development.

Pragmatically we have successfully adopted the simple approach of using established CR programs rather that developing a TAVI-specific programme which would be expensive and logistically challenging. We plan to continue this approach for a subsequent clinical outcome powered trial.

Participating centres will be required to have a centre-based CR programme preferably with BACPR accreditation. We plan to deliver CR earlier post-TAVI and to increase the intensity and duration for the CR to three sessions per week for 12 weeks and starting earlier than day 30. Although we selected patients > 75 years of age, TAVI is now being undertaken in significant numbers of younger patients, often with complex comorbidities who may benefit from CR and we may need to consider reducing or omitting the age cut-off.

The 6-month follow-up period was dictated by financial and resource implications in this pilot however in a subsequent funded clinically powered study we propose a 1-year follow-up period.

Considering our experience of the complex issues around frailty independence and emotional assessment in this cohort, we are exploring other HRQoL measures including KCCQ as the primary outcome measure. Secondary outcome measures will include exercise capacity (e.g. 6-min walk test), mortality, hospital readmission rates, mortality, psychological well-being (e.g. HADS), generic HRQoL (SF-12 and EQ-5D-5L), physical activity (accelerometer) and health care utilisation.

## Conclusions

In this study, we have established the feasibility of recruiting patients into a randomised trial of CR following the TAVI procedure in a single centre. The centre operates similar pathways and operating procedures to other UK centres such that the conclusions are likely to generalisable. We will use the findings of this pilot trial to plan the design and funding of an outcome powered UK multicentre RCT to inform the provision of CR and support guideline development to optimise health-related quality of life outcomes in this vulnerable population.

## Additional files

Additional file 1: CONSORT Checklist. (PDF 358 kb)

Additional file 2: Trial Protocol. (PDF 728 kb)

Additional file 3: Patient Characteristics. (PDF 224 kb)

Additional file 4: Patient Outcomes. (PDF 228 kb)

Additional file 5: Patient HRQoL Outcomes. (PDF 319 kb)
