# Supplementary material for: Cardiac rehabilitation to improve health-related quality of life following trans-catheter aortic valve implantation: a randomised controlled feasibility study: RECOVER–TAVI Pilot, ORCA 4, For the Optimal Restoration of Cardiac Activity Group
Source: Pilot Feasibility Stud. 2018 Dec 13;4:185. doi: 10.1186/s40814-018-0363-8 (PMC6293531; doi:10.1186/s40814-018-0363-8)
Supplement: Supplementary file 2 — Trial Protocol. (PDF 728 kb) [file 40814_2018_363_MOESM2_ESM.pdf]

Does Cardiac REhabilitation improve functional, independence, frailty and emotional outCOmes following Trans Catheter Aortic ValvE Replacement?

Short Study Title/Acronym: RECOVER – TAVR Pilot

REC Reference: 16/OO/0687 London – Queens Square

CHIEF INVESTIGATOR:

Mrs Paula Rogers

Research Nurse Manager

Royal Brompton and Harefield NHS Foundation Trust

Hill End Road, Harefield, Uxbridge, Middlesex

UB9 6JH

Phone: 01895 – 823737 Bleep 6085 or 07753-766189

Email: p.rogers@rbht.nhs.uk

Fax: TBC

SPONSOR REPRESENTATIVE:

Dr Ginette Hoare

Royal Brompton and Harefield NHS Foundation Trust (RB&HFT)

Royal Brompton Hospital (RBH)

Research Office

Chelsea Wing, Level 2

Sydney Street

London SW3 6NP

Phone: 0207 352 8121 ext. 2610

Email: g.hoare@rbht.nhs.uk

Fax: 020 8725 0794

Information in this protocol is confidential and should not be disclosed, other than to those directly involved in the execution or the ethical review of the study, without written authorisation from RB&HFT Research Office.

Table of contents

1. LIST OF ABBREVIATIONS 4
2. STUDY PERSONNEL AND FACILITIES 5
3. STUDY SYNOPSIS 6
4. INTRODUCTION 7
4.1 BACKGROUND 7
4.2 PRE-CLINICAL DATA/CLINICAL DATA 8
4.3 STUDY RATIONALE AND RISK/BENEFIT ANALYSIS 8
4.4 MANAGEMENT OF POTENTIAL STUDY RISKS 8
5. STUDY OBJECTIVES 9
5.1 PRIMARY OBJECTIVE 9
5.2 SECONDARY OBJECTIVES 9
6. TRIAL DESIGN 9
6.1 OVERALL DESIGN 9
6.2 TREATMENT AND RATIONALE 9
6.3 SCHEMATIC OF TRIAL DESIGN 9
7. ELIGIBILITY CRITERIA 10
7.1 INCLUSION CRITERIA 10
7.2 EXCLUSION CRITERIA 10
7.3 DISCONTINUATION/WITHDRAWAL OF PARTICIPANTS AND STOPPING RULES 10
8. SUBJECT/PATIENT RECRUITMENT PROCESS 10
9. STUDY PROCEDURES 10
9.1 INFORMED CONSENT 10
9.2 RANDOMISATION PROCEDURE 11
9.3 EMERGENCY UNBLINDING 11
10. STUDY ASSESSMENTS 11
10.1 SCREENING ASSESSMENTS 11
10.2 BASELINE ASSESSMENTS 11
10.3 TREATMENT PROCEDURE 12
10.4 SUBSEQUENT ASSESSMENTS 12
10.5 SUMMARY CHART OF STUDY ASSESSMENTS 12
11. METHODS 12
11.1 Laboratory Procedures 12
11.2 Radiology or any other procedure(s) – *Please define applicable procedure(s) in this heading* 12
11.3 DEFINITION OF THE END OF TRIAL 13
12. SAFETY REPORTING 13

12.1 DEFINITIONS 13

12.2 RECORDING ADVERSE EVENTS (AES) ERROR! BOOKMARK NOT DEFINED.

12.3 REPORTING SAES

12.4 THE TYPE AND DURATION OF THE FOLLOW-UP OF SUBJECTS AFTER AES

12.5 PREGNANCY

12.6 ANNUAL PROGRESS REPORTS (APRS)

13.2 DATA COLLECTION TOOL

13.3 DATA HANDLING AND ANALYSIS

13.4 ARCHIVING ARRANGEMENTS

14. STATISTICAL DESIGN

14.1 SAMPLE SIZE AND RECRUITMENT

14.2 ENDPOINTS

14.2.1 Primary endpoints

14.2.2 Secondary endpoints

14.3 STATISTICAL ANALYSIS PLAN

14.3.1 Primary endpoint analysis

14.3.2 Secondary endpoint analysis

14.4 RANDOMISATION

14.5 INTERIM ANALYSIS (IF APPLICABLE)

14.6 OTHER STATISTICAL CONSIDERATIONS

15. COMMITTEES IN INVOLVED IN THE STUDY

16. MONITORING AND AUDITING

16.1 DIRECT ACCESS TO SOURCE DATA

17. ETHICS AND REGULATORY REQUIREMENTS

18. FINANCE

19. INSURANCE AND INDEMNITY

20. PUBLICATION POLICY

21. STATEMENT OF COMPLIANCE

22. LIST OF PROTOCOL APPENDICES

23. REFERENCES

1. LIST OF ABBREVIATIONS

AE	Adverse Event
AR	Adverse Reaction
ASR	Annual Safety Report
CI	Chief Investigator
CRF	Case Report Form
DMC	Data Monitoring Committee
EXACT	Exacerbations of Chronic Pulmonary Disease Tool
GAfREC	Governance Arrangements for NHS Research Ethics
GCP	Good Clinical Practice
GMP	Good Manufacturing Practice
ICF	Informed Consent Form
ISF	Investigator Site File
ISRCTN	International Standard Randomised
MUST	Malnutrition Universal Screening Tool
NHS R&D	National Health Service Research & Development
NIMP	Non- Investigational Medicinal Product
PI	Principal Investigator
PIS	Participant Information Sheet
QA	Quality Assurance
QC	Quality Control
RCT	Randomised Control Trial
REC	Research Ethics Committee
SAR	Serious Adverse Reaction
SAE	Serious Adverse Event
SDV	Source Document Verification
SOP	Standard Operating Procedure
SmPC	Summary of Product Characteristics
SSA	Site Specific Assessment
TMG	Trial Management Group
TSC	Trial Steering Committee

2. STUDY PERSONNEL AND FACILITIES

Principal Investigator (PI): Paula Rogers

Royal Brompton and Harefield NHS Foundation Trust
Harefield Hospital
Hill End Road
Middx, UB9 6JH
E-mail: p.rogers@rbht.nhs.uk
Phone: 01895-823737 Bleep – 6085 or 07753-766189

Statistician: **Mr Winston Banya**

Royal Brompton and Harefield NHS Foundation Trust
E-mail: w.banya@rbht.nhs.uk
Phone: 0207-3528121

Medical Expert: **Dr Miles Dalby**

Royal Brompton and Harefield NHS Foundation Trust
Harefield Hospital
Hill End Road
Middx, UB9 6JH

E-mail: m.dalby@rbht.nhs.uk
Phone: 01895-823737 Ext 5990

Expert Advisor: **Professor Rod Taylor MSc, PhD**
Chair of Health Services Research, Director of Exeter Clinical Trials Unit & NIHR Senior Investigator

Institute of Health Research
University of Exeter Medical School
South Cloisters
St Lukes Campus, Heavitree Road
Exeter EX1 2LU
Office: + 44 (0)1392 726053

Email: r.taylor@exeter.ac.uk

3. STUDY SYNOPSIS

**Full study title:**	A pilot study of the impact of cardiac rehabilitation to improve functional, independence, frailty and emotional outcomes following Trans Catheter Aortic Valve Replacement (TAVR).
**Short study title:**	RECOVER – TAVR Pilot
**Study R&D number:**	
**Study drug (s):**	N /A
**Chief Investigator:**	Paula Rogers
**Medical condition/disease under investigation:**	Aortic Stenosis
**Study duration:**	12 months
**Clinical phase:**	Pilot Study
**Primary Objective:**	1) To assess the feasibility of conducting additional functional, independence, frailty and emotional tests in the in- patient and out-patient setting.
**Secondary Objective:**	1) To assess the uptake of patients willing to undertake cardiac rehabilitation.<br>2) To assess the feasibility of providing cardiac rehabilitation in this patient population.<br>3) To assess the impact of cardiac rehabilitation versus standard of care in patients who have received TAVI.<br>4) To assess if the effect of cardiac rehabilitation is sustained at 6 months post TAVI.
**Study population:**	30 patients who have been accepted for Trans Catheter Aortic Valve Replacement
**Methodology:**	Randomised Controlled Trial

<table>
<tr><td></td><td></td></tr>
<tr><td rowspan="2">Eligibility criteria:</td><td>Inclusion criteria:
Severe symptomatic aortic stenosis accepted for Trans Catheter Aortic Valve Replacement
Age ≥75 years;
Participant able and willing to give written informed consent;
Participant able (in the Investigator's opinion) and willing to comply with all study requirements.</td></tr>
<tr><td>Exclusion criteria:
Subjects may not enter the study if ANY of the following apply:
Intervention deemed inappropriate due to co-morbidity or frailty;
Life expectancy less than one year due to co-morbidity;
Previous AVR or TAVI;
Predominant aortic regurgitation (AR).</td></tr>
<tr><td colspan="2">Study treatment: (i.e. dose and mode of the study drug administration if applicable):</td></tr>
<tr><td colspan="2">15 patients will be randomised to receive a programme of cardiac rehabilitation, and 15 patients will receive standard care.</td></tr>
</table>

4. INTRODUCTION

4.1 BACKGROUND

Aortic stenosis is the most common form of valvular heart disease in the elderly and is associated with high morbidity and mortality once cardiac symptoms develop (1). In patients who are at high risk for serious complications during or after surgery, Transcatheter Aortic Valve Implantation (TAVI) has been shown to result in reductions in mortality and improvement in quality of life compared with medical therapy (2, 3).
Due to the ageing and increasingly complex nature of patients with aortic stenosis, frailty and functional assessment has become a high-priority theme within patient management. Frailty is a

term widely used to describe a multidimensional syndrome of loss of reserves (energy, physical ability, cognition, health) that gives rise to vulnerability (4). Several publications deal with the impact of frailty on mortality and morbidity in the elderly population (5). Functional status is evaluated by the ability to undertake Activities of Daily Living (ADL) and it has been demonstrated that frailty and the onset of dependence in ADL are strongly associated (6).

4.2 Pre-clinical data/clinical data

Cardiac rehabilitation is a complex intervention offered to patients with heart disease and includes components of health education, advice on cardiovascular risk reduction, physical activity and stress management. Cardiac rehabilitation and physical activity are recommended treatments after cardiac valve surgery and positively improves morbidity, exercise capacity and quality of life or emotional well-being. The National Institute for Healthcare Excellence and The Department of Health and wider European guidelines agree that patients who have received heart valve replacements will benefit from cardiac rehabilitation (7).

4.3 Study Rationale and risk/benefit analysis

A literature search has not revealed any publications which specifically outline the impact of receiving a programme of cardiac rehabilitation, following TAVR and this study aims to address that question. The ethical dilemma of conducting research in elderly patients who have undergone TAVR can be associated with clinical trials in other elderly populations. The dilemma consists of the need to develop new and better treatment options for a particular group of patients whilst protecting the patient from harm. We anticipate that this study will allow us to understand the feasibility of undertaking this study in a group of patients who have agreed to participate in a research study prior to the TAVR procedure. The pilot study will help us to understand the logistics of providing a cardiac rehabilitation programme for patients and the data we generate will allow us to understand if there are any benefits related to functional, independence, frailty and emotional domains for this patient group. The outcomes from this pilot study may allow us to plan future studies with the aim of developing appropriate guidelines related to the provision of cardiac rehabilitation for this patient group.

4.4 Management of potential study risks

Patients who are eligible for TAVR, as determined by the multi-disciplinary team, will be seen in the out-patient clinic or in-patient area by the Research Nurse Manager or delegated research nurse. Full informed consent will be obtained before any research intervention is undertaken. Functional, independence, frailty and emotional assessment will include 6 minute walk teat, 4m gait speed walk, hand grip strength, questionnaires and cognitive assessment (Appendix enclosed).

Patients will undergo TAVR as per routine practice and they will be discharged when safe to do so. The research nurse team will collect clinical data during the admission. All patients will attend clinic for review at 4 weeks post implant, research consent will be reaffirmed and the patients will be randomised to receive a cardiac rehabilitation programme or standard of care.

A personalised programme of cardiac rehabilitation will be developed by expert physiotherapists. Patients in the cardiac rehab group will be assessed for the cardiac rehabilitation prior to any intervention taking place. Patients are monitored during exercise and full emergency equipment is available should there be an emergency.

Patients who are in the standard of care group will have access to the TAVR nurse specialist as well as the designated Consultant and research nurse team to ask any advice at any time.

5. STUDY OBJECTIVES

5.1 PRIMARY OBJECTIVE

1) To assess the feasibility of conducting additional functional, independence, frailty and emotional tests in the in- patient and out-patient setting.

5.2 SECONDARY OBJECTIVES

1) To assess the uptake of patients willing to undertake cardiac rehabilitation.
2) To assess the feasibility of providing cardiac rehabilitation in this patient population.
3) To assess the impact of cardiac rehabilitation versus standard of care in patients who have received TAVR.
4) To assess if the effect of cardiac rehabilitation is sustained at 6 months post TAVR.

6. TRIAL DESIGN

6.1 OVERALL DESIGN

The study will be a randomised controlled design. A randomisation table will be provided by the Trust statistician and be accessed via the internet. 15 patients will receive a programme of cardiac rehabilitation and 15 patients will receive normal care.

6.2 TREATMENT AND RATIONALE

The rationale for this study is related to the lack of published evidence related to the provision of cardiac rehabilitation for elderly patients who have received TAVR as an active treatment for aortic stenosis. We are very keen to understand this experience within our own institution. This pilot study will allow the research team to capture data related to the feasibility of undertaking such a study in this patient population. There is value in robustly assessing patients for the parameters of functional, independence, frailty and emotional parameters before TAVR is undertaken. Repeating the measures post –TAVR and randomising half of the patients to receive a programme of cardiac rehabilitation will allow us to understand the impact of cardiac rehabilitation compared to routine standard of care, during the follow up period. The pilot data will inform a future randomised controlled trial which may include UK wide TAVR centres, if research study funding can be secured.

6.3 SCHEMATIC OF TRIAL DESIGN

Please see the schematic table enclosed.

7. ELIGIBILITY CRITERIA

7.1 INCLUSION CRITERIA

- Severe symptomatic aortic stenosis accepted for Trans Catheter Aortic Valve Replacement
- Age ≥75 years;
- Participant able and willing to give written informed consent;
- Participant able (in the Investigator's opinion) and willing to comply with all study requirements.

7.2 EXCLUSION CRITERIA

- Subjects may not enter the study if ANY of the following apply:
- Intervention deemed inappropriate due to co-morbidity or frailty;
- Life expectancy less than one year due to co-morbidity;
- Previous AVR or TAVI;
- Predominant aortic regurgitation (AR).

7.3 DISCONTINUATION/WITHDRAWAL OF PARTICIPANTS AND STOPPING RULES

Should participants wish to withdraw from the trial, data collected to date will be kept and may be used for research purposes with the patient's agreement. All reasons for voluntary withdrawal from the study will be documented.

8. SUBJECT/PATIENT RECRUITMENT PROCESS

Patient recruitment at a site will only commence once the trial team has ensured that the following approval/essential documents are in place:

1. HRA Approval,
2. Confirmation of Capacity and Capability
3. Local Site Delegation of Duties and Signature Log is completed.

All subjects who wish to enter the study will be fully screened and consented by the Chief Investigator (CI), or one of the delegated individuals involved in the study as the Principal Investigator (PI).

Eligible patients will be approached about the study during a routine out-patient appointment or at the time of their admission, the day before the TAVR procedure. Patients scheduled for TAVR will be aware of the study via a telephone call prior to their admission, for those patients who do not attend the out-patient department. The patient information sheet will be posted to patients who express an interest to receive more information.

Study procedures

9.1 INFORMED CONSENT

Informed consent will be obtained by the Principal Investigator (PI) and/or a nominated deputy as recorded on Sponsor's Delegation of Responsibilities Log. Only those members of the study team who have clinical responsibility for the care of patients under the care of the general medical service will be permitted to undertake informed consent. All individuals taking informed consent will have received consent training.

Consent to enter this study will be obtained after a full account has been provided of its nature, purpose, risks, burdens and potential benefits, and the patient has had the opportunity to deliberate. The patient will be allowed to specify the time they wish to spend deliberating, usually up to 24 hours.

Periods shorter than 24 hours will be permitted if the patient feels that further deliberation will not lead to a change in their decision, and provided the person seeking consent is satisfied that the patient has fully retained, understood and deliberated on the information given. This provision has been made with the support of our patient advisory group.

Likewise, periods longer than 24 hours will be permitted should the patient request this. The Investigator or designee will explain that the patients are under no obligation to enter the trial and that they can withdraw at any time during the trial, without having to give a reason.

A copy of the signed Informed Consent Form (ICF) along with a copy of the most recent approved Patient Information Sheet (PIS) will be given to the study participant. The original signed consent form will be retained at the study site (one filed in the medical notes and one field in the Trial Master File (TMF)). A copy of the consent form will also be given to the patient.

9.2 RANDOMISATION PROCEDURE

1:1 randomisation will be used to determine which patients will receive a programme of cardiac rehabilitation. The simple randomisation table will be created by the Trust statistician and will be undertaken by a member of the research team, in normal working hours.

9.3 EMERGENCY UNBLINDING

This section does not apply. It is not possible to blind the randomisation group allocated.

9. STUDY ASSESSMENTS

10.1 SCREENING ASSESSMENTS

Patients with aortic stenosis are discussed at the aortic MDT meeting to determine suitability for Aortic Valve Replacement, TAVR or medical management. Patients who are eligible for TAVR will be highlighted to the research nurse team and approached via telephone in the first instance, to inform the patients about the study. Patients will then be seen in the out-patient clinic or the ward area.

10.2 BASELINE ASSESSMENTS

10.3 Baseline assessment will include provision of fully informed consent. Patients will undergo a 6 minute walk test, frailty assessment and complete the questionnaires enclosed in the appendix. TAVR will be undertaken as usual practice and patients will be discharged home when safe to do so. The research nurse team will collect clinical details of the hospital admission period.

10.4 TREATMENT PROCEDURE

Patients will return to the out-patient clinic at 4 weeks post – TAVR and research consent will be re-affirmed. Patients will be randomised to receive a programme of cardiac rehabilitation or routine care at this point. Patients randomised to receive cardiac rehabilitation will meet the cardiac rehab team and undergo baseline assessment for the programme. This involves recording height, weight, blood pressure, oxygen saturation and heart rate and rhythm. An individualised programme will be established to meet the needs of each patient. The patients will undergo a 6 week programme of cardiac rehab. Patients who are not allocated to the intervention group will not receive a cardiac programme and will have access to the TAVR nurse specialist team as would be usual care.

10.5 SUBSEQUENT ASSESSMENTS

All patients will return to the out-patient clinic at 3 and 6 months post TAVR and undergo the same assessment as the baseline.

10.6 SUMMARY CHART OF STUDY ASSESSMENTS

Please see the schematic table enclosed.

10. METHODS

11.1 Laboratory Procedures

This section does not apply.

11.2 Radiology or any other procedure(s)

- The TAVR procedure uses radiation. This will be declared on the Research Ethics application via IRAS but radiation will not be part of this protocol.

- ***Techniques and Interventions***

The aim of cardiac rehabilitation is to help patients recover and to get back to a full life following cardiac procedures. It aims to promote health and well-being once patients have recovered from the TAVR. Cardiac rehabilitation is an established intervention for patients who have suffered a heart attack or undergone heart surgery. It is currently not offered routinely to patients who have undergone TAVR. Cardiac rehabilitation includes components of health education, advice on cardiovascular risk reduction, physical activity and stress management. Cardiac rehabilitation will be delivered by trained professionals, familiar with working with patients with heart failure and multiple co-morbidities.

- ***Tools***

Questionnaires used in this study have been used by other research groups and are enclosed as an appendix.

- ***Study Drugs***

Not applicable to this study.

11.3 Definition of the End of Trial

The study will be complete when we have recruited 30 patients who are receiving TAVR as part of clinical management for their aortic stenosis. All 30 patients will be followed for 6 months beyond the TAVR procedure. The end of trial is defined as the last patient reaching the 6 months follow up time point.

11. SAFETY REPORTING

Adverse Event (AE) — any untoward medical occurrence in a patient or clinical trial subject who is administered a treatment and which does not necessarily have a causal relationship with this treatment (*i.e.* any unfavourable or unintended change in the structure (signs), function (symptoms), or chemistry (lab data) in a subject to whom a treatment/study procedure has been administered, including occurrences unrelated to that product/procedure/device).

Serious Adverse Event (SAE) – is defined as an untoward occurrence that:

- Results in death; or
- Is life-threatening (places the subject, in the view of the Investigator, at immediate risk of death)
- Requires hospitalization or prolongation of existing hospitalization (hospitalisation is defined as an inpatient admission, regardless of length of stay; even if it is a precautionary measure for observation; including hospitalisation for an elective procedure, for a pre-existing condition)
- Results in persistent or significant disability or incapacity (substantial disruption of one's ability to conduct normal life functions)
- Consists of a congenital anomaly or birth defect (in offspring of subjects or their parents taking the study drug regardless of time of diagnosis)
- Is otherwise considered medically significant by the investigator.

Important medical events that may not be immediately life-threatening or result in death or hospitalisation but may jeopardise the subject or may require intervention to prevent one of the outcomes listed in the definition of serious will also be considered serious.

12.2 Recording Adverse Events (AEs)

All Adverse Events will be recorded in the hospital notes and Case Report Form (CRF).

If the Investigator suspects that the disease has progressed faster due to the administration of the study treatment/procedure, then he/she will report this as an unexpected adverse event to the Sponsor and the main REC as detailed in Section 12.3.

12.3 Assessment and Reporting SAEs

Principal Investigator (PI) at all sites must report all SAEs to the Chief Investigator (CI) or a delegated individual in the research team. The CI and his research team at RBH are responsible for reporting events to the Research Office immediately and/or within 24 hours of becoming aware of the event using the Sponsor's SAE Reporting Form.

Classification and causality of Adverse Events (AEs) will be conducted by local PIs and reviewed by CI. The CI cannot downgrade the site PI's classification and if there is disagreement which cannot be resolved during formal discussion then the assessment of the site PI will be accepted. The CI, can however, upgrade the seriousness of an event without consultation with the site PI.

All Adverse Events that are to be reported to the Research Office must be signed and dated and completed by the Investigator.

Information can be submitted in electronic format:

- Email: safetyreporting@rbht.nhs.uk or
- Fax: 0207 351 8829.

The research team also has the responsibility to report SAEs occurring in a certain period (28 days) after a patient completes the trial. Any SAEs reported to the Investigators during this phase must be documented in the patient's medical notes and submitted via an SAE form

The Research Ethics Committee (REC) that gave a favorable opinion of the study (the 'main REC') should be informed where in the opinion of the CI/PI the event was:

'related': that is, it resulted from administration of any of the research procedures; and

'Unexpected': that is, the type of event is not listed in the protocol as an expected occurrence.

Reports of related and unexpected SAEs should be submitted within 15 days of the CI/PI becoming aware of the event, using the form which can be found on the Health Research Authority's website. The form should be completed in typescript and signed by the Chief Investigator.

12.4 The type and duration of the follow-up of subjects after AEs

Patients who experience an SAE will be followed up for up to a month after the last observation. Adverse Events be recorded and reported up to one week after the last observation has been made.

12.5 Pregnancy

Pregnant patients will not be recruited into this study.

12.6 Annual Progress Reports (APRs)

The Chief Investigator will prepare the APR for the study. It will be reviewed by the RO and sent to the main REC by the CI within 30 days of the anniversary date on which the

favourable opinion was given by the main REC, and annually until the study is declared ended.

12.7 Reporting Urgent Safety Measures

The Sponsor and/or the Investigator may take appropriate urgent safety measures in order to protect the subjects of a clinical study against any immediate hazard to their health or safety. If safety measures are taken, the main REC approval is not required before the measure is taken.

The Investigator will immediately and in any event no later than 3 days from the date the measures are taken, give written notice to the main REC and the study Sponsor of the measures taken and the circumstances giving rise to those measures.

In order to prevent any delays in the reporting timelines the Sponsor has delegated this responsibility to the CI/PI. Therefore the CI/PI must report any urgent safety measures to the main REC directly, and in parallel to the Sponsor. The REC coordinator will acknowledge receipt of urgent safety measures within 30 days.

12. DATA MANAGEMENT AND QUALITY ASSURANCE

13.1 Confidentiality

All data will be handled in accordance with the Data Protection Act 1998, NHS Caldecott Principles, The Research Governance Framework for Health and Social Care, 2nd Edition (2005), and the condition of the main REC approval.

The Case Report Forms (CRFs) will not bear the subject's name or other personal identifiable data. The subject's initials, Date of Birth (DOB) and trial Identification Number (ID), will be used for identification.

13.2 Data collection tool

Case Report Forms (CRF) will be designed by the CI and the final version will be reviewed and discussed with the study Sponsor. All data will be entered legibly in black ink with a ball-point pen. If the Investigator makes an error, it will be crossed through with a single line in such a way to ensure that the original entry can still be read. The correct entry will then be clearly inserted. The amendment will be initialled and dated by the person making the correction immediately. Overwriting or use of correction fluid will not be permitted.

It is the Investigator's responsibility to ensure the accuracy of all data entered and recorded in the CRFs. The Delegation of Responsibilities Log will identify all trial personnel responsible for data collection, entry, handling and managing the database.

Interviews and focus groups will be tape-recorded and this will be made explicit in the patient information sheet. The patient narratives will be reviewed by at least two members of the study group.

13.3 Data handling and analysis

Patient participants will be allocated a study number. A database to collect the clinical data and responses from questionnaires will be designed. The database will be accessed via Trust computers with a log in for the research nurse team.

13.4 Archiving Arrangements

The study documents (including the Trial Master File (TMF), Case Report Forms (CRFs), Informed Consent Forms along with the trial database) will be kept for a minimum of five years. They will be stored in locked offices within the Royal Brompton and Harefield NHS

Foundation Trust (RB&HFT). The CI is responsible for the secure archiving of trial documents. The trial database will also be kept electronically on the RB&HFT computer network, for a minimum of five years.

The approved repository for longer retention of local materials for studies that involve RB&HFT patients is Box-It Storage UK. The study documentation will be prepared for archiving by the research team in line with the Research Office Archiving SOP and the transfer will be arranged by the Research Office.

13. STATISTICAL DESIGN

14.1 SAMPLE SIZE AND RECRUITMENT

This is a pilot study to determine the logistics of conducting such a study in an elderly population. Statistical support will be provided by the Trust statistician, Mr Winston Banya. The sample size is 30.

14.2 ENDPOINTS

14.2.1 Primary endpoints

1) To assess the feasibility of conducting additional functional, independence, frailty and emotional tests in the in- patient and out-patient setting.

14.2.2 Secondary endpoints

2) To assess the uptake of patients willing to undertake cardiac rehabilitation.
3) To assess the feasibility of providing cardiac rehabilitation in this patient population.
4) To assess the impact of cardiac rehabilitation versus standard of care in patients who have received TAVI.
5) To assess if the effect of cardiac rehabilitation is sustained at 6 months post TAVI.

14.2 STATISTICAL ANALYSIS PLAN

Categorical data will be presented as number and proportion and comparisons done using the chi-squared or Fishers exact test. Numeric data will be presented as mean ± SD for normally distributed data or median (Interquartile range) for non-normally distributed data. Comparisons of numeric data will be done using the 2 sample independent t test or the Wilcoxon rank-sum (Mann-Whitney) test. All analyses will be done according to Intention-to-treat and all tests will

be 2 sided with significance set at $p < 0.05$. No imputation of missing data will be done and effort will be made to ensure that the data are as complete as possible. No multiple comparisons will be done. All the analyses will be done using the statistical software Stata version 14.1. A detailed CONSORT diagram on the recruitment and management of the patients from screening to analysis will be presented.

14.3.1 Primary endpoint analysis

Data collected on additional functional, independence, frailty and emotional tests will be compared between the 2 groups using either the 2 sample independent t test or the Wilcoxon rank-sum test. The rate of recruitment per month will also be assessed against the target for that period.

4.4 MANAGEMENT OF POTENTIAL STUDY RISKS

Patients who are eligible for TAVI, as determined by the multi-disciplinary team, will be seen in the out-patient clinic or in-patient area by the Research Nurse Manager or delegated research nurse. Full informed consent will be obtained before any research intervention is undertaken. Functional, independence, frailty and emotional assessment will include 6 minute walk teat, 4m gait speed walk, hand grip strength, questionnaires and cognitive assessment (Appendix enclosed).

Patients will undergo TAVI as per routine practice and they will be discharged when safe to do so. The research nurse team will collect clinical data during the admission. All patients will attend clinic for review at 4 weeks post implant, research consent will be reaffirmed and the patients will be randomised to receive a cardiac rehabilitation programme or standard of care.

A personalised programme of cardiac rehabilitation will be developed by expert physiotherapists. Patients in the cardiac rehab group will be assessed for the cardiac rehabilitation prior to any intervention taking place. Patients are monitored during exercise and full emergency equipment is available should there be an emergency.

5.1 PRIMARY OBJECTIVE

2) To assess the feasibility of conducting additional functional, independence, frailty and emotional tests in the in- patient and out-patient setting.

5.2 SECONDARY OBJECTIVES

5) To assess the uptake of patients willing to undertake cardiac rehabilitation.
6) To assess the feasibility of providing cardiac rehabilitation in this patient population.
7) To assess the impact of cardiac rehabilitation versus standard of care in patients who have received TAVI.
8) To assess if the effect of cardiac rehabilitation is sustained at 6 months post TAVI.

14.3 Statistical analysis plan

Categorical data will be presented as number and proportion and comparisons done using the chi-squared or Fishers exact test. Numeric data will be presented as mean ± SD for normally distributed data or median (Interquartile range) for non-normally distributed data. Comparisons of numeric data will be done using the 2 sample independent t test or the Wilcoxon rank-sum (Mann-Whitney) test. All analyses will be done according to Intention-to-treat and all tests will be 2 sided with significance set at $p < 0.05$. No imputation of missing data will be done and effort will be made to ensure that the data are as complete as possible. No multiple comparisons will be done. All the analyses will be done using the statistical software Stata version 14.1. A detailed CONSORT diagram on the recruitment and management of the patients from screening to analysis will be presented.

14.3.1 Primary endpoint analysis

Data collected on additional functional, independence, frailty and emotional tests will be compared between the 2 groups using either the 2 sample independent t test or the Wilcoxon rank-sum test. The rate of recruitment per month will also be assessed against the target for that period.

14.3.2 Secondary endpoint analysis

The same method of analysis of the primary endpoint will be used in the analysis of the secondary endpoints.

14.4 Randomisation

A computer generated block randomisation schedule will be done using the software Stata version 14.1. All participants will be randomly assigned to one of 2 groups with equal allocation between the 2 groups.

14.5 Interim analysis (if applicable)

Not Applicable

14.6 Other statistical considerations

Not Applicable

14. COMMITTEES IN INVOLVED IN THE STUDY

1. **Trial Management Group (TMG)** – will include those individuals responsible for the day-to-day management of the trial, such as the CI, the medical supervisor, statistician, trial

manager, research nurses, and data manager. The role of the group is to monitor all aspects of the conduct and progress of the trial, ensure that the protocol is adhered to and take appropriate action to safeguard participants and the quality of the trial itself.

15. MONITORING AND AUDITING

The requirement for study monitoring or audit will be based on the internal Research Office risk assessment procedure and applicable Standard Operating Procedures (SOPs). It is the responsibility of the RO to determine the monitoring risk assessment and explain the rationale to the study research team.

Study monitoring and/or audit will be discussed with the CI before arrangements are made to conduct the visit.

16.1 DIRECT ACCESS TO SOURCE DATA

The Investigator(s)/institution(s) will permit trial-related monitoring, audits, REC review, and regulatory inspection(s), providing direct access to source data/documents. Trial participants are informed of this during the informed consent discussion. Participants will consent to provide access to their medical notes.

16. ETHICS AND REGULATORY REQUIREMENTS

The Sponsor will ensure that the trial protocol, Patient Information Sheet (PIS), Informed Consent Form (ICF), GP letter and submitted supporting documents have been approved by the HRA, prior to any patient recruitment taking place. The protocol and all agreed substantial protocol amendments, will be documented and submitted for ethical approval prior to implementation.

Before site(s) can enrol patients into the trial, the PI must receive confirmation of capacity and capability from the Trust Research & Development (R&D). It is the responsibility of the PI at each site to ensure that all subsequent amendments gain the necessary approval. This does not affect the individual clinician's responsibility to take immediate action if thought necessary to protect the health and interest of individual patients.
Within 90 days after the end of the trial, the CI and Sponsor will ensure that the main REC is notified that the trial has finished. If the trial is terminated prematurely, those reports will be made within 15 days after the end of the trial.

The CI will supply a final summary report of the clinical trial to the main REC and the Sponsor in parallel within one year after the end of the trial.

17. FINANCE

The study is being funded via the Royal Brompton and Harefield NHS Foundation Trust, Cardiovascular and Respiratory Biomedical Research Units. Following a pump priming call, the study team has secured £9000 to cover the costs of cardiac rehabilitation, patient travel expenses and monies towards a database.

18. INSURANCE AND INDEMNITY

NHS bodies are liable for clinical negligence and other negligent harm to individuals covered by their duty of care. NHS Institutions employing researchers are liable for negligent harm caused by the design of studies they initiate. The provision of such indemnity for negligent harm should be stated to the participant.

19. PUBLICATION POLICY

Data ownership rights will lie with the institution.

20. STATEMENT OF COMPLIANCE

The trial will be conducted in compliance with the protocol, Sponsor's Standard Operating Procedures (SOPs), GCP and the applicable regulatory requirement(s).

The study conduct shall comply with all relevant laws of the EU if directly applicable or of direct effect and all relevant laws and statutes of the UK country in which the study site is located including but not limited to, the Human Rights Act 1998, the Data Protection Act 1998, the Medicines Act 1968, and with all relevant guidance relating to medicines and clinical studies from time to time in force including, but not limited to, the ICH GCP**,** the World Medical Association Declaration of Helsinki entitled 'Ethical Principles for Medical Research Involving Human Subjects' (2008 Version), the NHS Research Governance Framework for Health and Social Care (Version 2, April 2005).

This study will be conducted in compliance with the protocol approved by the main REC and according to RGF standards. No deviation from the protocol will be implemented without the prior review and approval of the Sponsor and the main REC except where it may be necessary to eliminate an immediate hazard to a research subject. In such case, the deviation will be reported to the Sponsor and the main REC as soon as possible.

21. LIST OF PROTOCOL APPENDICES

Appendix 1 Summary Chart of Study Assessments
Appendix 2 Questionnaires and Frailty assessment templates.

REFERENCES

1.Bonow RO, Carabello BA, Chatterjee K, et al. 2008 focused update incorporated into the ACC/AHA 2006 guidelines for the management of patients with valvular heart disease: a report of the American College of Cardiology/American Heart Association Task Force on Practice Guidelines (Writing Committee to revise the 1998 guidelines for the management of patients with valvular heart disease). Endorsed by the Society of Cardiovascular Anesthesiologists, Society for Cardiovascular Angiography and Interventions, and Society of Thoracic Surgeons. J Am Coll Cardiol. 2008; 52:e1–142. [PubMed: 18848134]

2.Leon MB, Smith CR, Mack M, et al. Transcatheter aortic-valve implantation for aortic stenosis in patients who cannot undergo surgery. N Engl J Med. 2010; 363:1597–607. [PubMed: 20961243]

3.Reynolds MR, Magnuson EA, Lei Y, et al. Health-related quality of life after transcatheter aortic valve replacement in inoperable patients with severe aortic stenosis. Circulation. 2011; 124:1964–72. [PubMed: 21969017].

4.Rockwood K[1], Song X, MacKnight C, Bergman H, Hogan DB, McDowell I, Mitnitski A. A global clinical measure of fitness and frailty in elderly people. 4. CMAJ. 2005 Aug 30;173(5):489-95.

5.Inouye SK, Peduzzi PN, Robison JT, Horwitz RI, Concato J. Importance of functional measures in predicting mortality among older hospitalized patients. JAMA. 1998;279: 1187-93.

6.Boyd CM, Xue QL, Simpson CF, Guralnik JM, Fried LP. Frailty, hospitalization, and progression of disability in a cohort of diabled older women. Am J Med. 2005;118: 1125-31.

7. Dalal HM, Doherty P, Taylor RS. Cardiac rehabilitation. BMJ. 2015:351: h5000 doi 10.1136.

Appendix 1

Summary chart of study Assessments (Template)

Please note that the summary chart below should only be used as guidance and ensure that the list of procedures in the chart as well as treatment and follow up timelines relate to procedures in your study.

**Study Procedures**	**Baseline**	**TAVR**	**Follow up** ***(4 Weeks)***	**Follow up** ***(3 Months)***	**Follow up** ***(6 Months)***
Informed consent	X		x		
Inclusion/exclusion criteria	X				
Medical history	X		x	x	x
Demographics	X		x	x	x
FRIED Frailty Score	X		x	x	x
Edmunton Frailty Score	X		x	x	x
6 Minute Walk Test	X		x	x	x
Nottingham ADL	X		x	x	x
HADS Questionnaire	X		x	x	x
Cardiac Rehab			x		
Concomitant Medication	x	x	x	x	x
