# Supplementary material for: Cardiac rehabilitation to improve health-related quality of life following trans-catheter aortic valve implantation: a randomised controlled feasibility study: RECOVER–TAVI Pilot, ORCA 4, For the Optimal Restoration of Cardiac Activity Group
Source: Pilot Feasibility Stud. 2018 Dec 13;4:185. doi: 10.1186/s40814-018-0363-8 (PMC6293531; doi:10.1186/s40814-018-0363-8)
Supplement: Supplementary file 3 — Patient Characteristics. (PDF 224 kb) [file 40814_2018_363_MOESM3_ESM.pdf]

RECOVER-TAVR ID
RT - 001
RT - 002
RT - 003
RT - 004
RT - 005
RT - 006
RT - 008
RT - 009
RT - 010
RT - 011
RT - 012
RT - 013
RT - 014
RT - 015
RT - 016
RT - 017
RT - 018
RT - 019
RT - 020
RT - 021
RT - 022
RT - 023
RT - 024
RT - 025
RT - 026
RT - 027
RT - 028
RT - 029
RT - 030
RT - 032
Note - Patient - RT - 007 and 031 did not receive TAVI, no infoflex data vailable

Type of TAVI procedure	Sex	Age	Diabetes	Smoking status
TAVI	2. Female	78	0. Not diabetic	0. Never smoked
TAVI	2. Female	80	0. Not diabetic	0. Never smoked
TAVI	1. Male	84	0. Not diabetic	1. Ex smoker
TAVI	1. Male	77	3. Diabetes (insulin)	0. Never smoked
TAVI	1. Male	90	0. Not diabetic	1. Ex smoker
TAVI	2. Female	85	0. Not diabetic	0. Never smoked
TAVI	2. Female	83	0. Not diabetic	0. Never smoked
TAVI	1. Male	85	0. Not diabetic	0. Never smoked
TAVI	1. Male	75	0. Not diabetic	1. Ex smoker
TAVI	2. Female	91	0. Not diabetic	1. Ex smoker
TAVI	1. Male	80	0. Not diabetic	1. Ex smoker
TAVI	2. Female	80	3. Diabetes (insulin)	1. Ex smoker
TAVI	1. Male	89	0. Not diabetic	1. Ex smoker
TAVI	1. Male	88	0. Not diabetic	0. Never smoked
TAVI	2. Female	82	1. Diabetes (dietary control)	0. Never smoked
TAVI	2. Female	85	0. Not diabetic	0. Never smoked
TAVI	2. Female	72	0. Not diabetic	0. Never smoked
TAVI	2. Female	80	0. Not diabetic	1. Ex smoker
TAVI	2. Female	82	0. Not diabetic	1. Ex smoker
TAVI	1. Male	78	0. Not diabetic	0. Never smoked
TAVI	1. Male	86	0. Not diabetic	1. Ex smoker
TAVI	1. Male	87	0. Not diabetic	1. Ex smoker
TAVI	1. Male	75	2. Diabetes (oral medicine)	1. Ex smoker
TAVI	2. Female	83	2. Diabetes (oral medicine)	1. Ex smoker
TAVI	1. Male	80	2. Diabetes (oral medicine)	0. Never smoked
TAVI	2. Female	77	0. Not diabetic	1. Ex smoker
TAVI	2. Female	83	0. Not diabetic	0. Never smoked
TAVI	1. Male	83	0. Not diabetic	1. Ex smoker
TAVI	1. Male	80	0. Not diabetic	1. Ex smoker
TAVI	1. Male	85	0. Not diabetic	0. Never smoked

Creatinine (mmol/l)	Previous MI and interval be	History of pulmonary diseas
87	0. No previous MI	0. No pulmonary disease
93	0. No previous MI	0. No pulmonary disease
123	0. No previous MI	1. COAD/emphysema
88	5. MI >90 days	0. No pulmonary disease
77	0. No previous MI	0. No pulmonary disease
131	0. No previous MI	0. No pulmonary disease
60	0. No previous MI	0. No pulmonary disease
133	0. No previous MI	1. COAD/emphysema
116	0. No previous MI	1. COAD/emphysema
86	0. No previous MI	0. No pulmonary disease
84	0. No previous MI	1. COAD/emphysema
56	5. MI >90 days	2. Asthma
157	0. No previous MI	1. COAD/emphysema
119	0. No previous MI	3. Other significant pulmonary disease
58	0. No previous MI	0. No pulmonary disease
92	0. No previous MI	0. No pulmonary disease
57	0. No previous MI	2. Asthma
72	5. MI >90 days	0. No pulmonary disease
63	0. No previous MI	0. No pulmonary disease
89	5. MI >90 days	0. No pulmonary disease
90	0. No previous MI	1. COAD/emphysema
118	0. No previous MI	0. No pulmonary disease
108	5. MI >90 days	0. No pulmonary disease
107	0. No previous MI	0. No pulmonary disease
100	0. No previous MI	0. No pulmonary disease
77	0. No previous MI	0. No pulmonary disease
64	0. No previous MI	0. No pulmonary disease
164	5. MI >90 days	0. No pulmonary disease
116	0. No previous MI	0. No pulmonary disease
84	0. No previous MI	0. No pulmonary disease

Pre-operative heart rhythm	Previous cardiac surgery
0. Sinus rhythm	0. No
0. Sinus rhythm	0. No
0. Sinus rhythm	0. No
4. LBBB,6. Paced rhythm	1. Previous CABG
1. Atrial fibrillation / flutter	1. Previous CABG
1. Atrial fibrillation / flutter	0. No
6. Paced rhythm	0. No
1. Atrial fibrillation / flutter,6. Paced rhythm	0. No
6. Paced rhythm	1. Previous CABG
1. Atrial fibrillation / flutter	0. No
1. Atrial fibrillation / flutter,6. Paced rhythm	1. Previous CABG
0. Sinus rhythm	1. Previous CABG
0. Sinus rhythm	0. No
6. Paced rhythm	0. No
1. Atrial fibrillation / flutter	0. No
1. Atrial fibrillation / flutter	0. No
1. Atrial fibrillation / flutter	0. No
0. Sinus rhythm	0. No
0. Sinus rhythm	0. No
1. Atrial fibrillation / flutter	0. No
0. Sinus rhythm	1. Previous CABG
1. Atrial fibrillation / flutter	0. No
1. Atrial fibrillation / flutter	1. Previous CABG
1. Atrial fibrillation / flutter	0. No
0. Sinus rhythm	0. No
0. Sinus rhythm	0. No
0. Sinus rhythm	0. No
0. Sinus rhythm	1. Previous CABG
0. Sinus rhythm	0. No
1. Atrial fibrillation / flutter	0. No

Previous PCI	BMI
2. Yes - as part of a staged or hybrid procedure	21.99
2. Yes - as part of a staged or hybrid procedure	33.04
2. Yes - as part of a staged or hybrid procedure	25.51
0. No	28.41
0. No	25.37
1. Yes - previous standalone PCI (NOT as part of staged or hybrid procedure)	29.42
0. No	24.88
0. No	27.1
0. No	32
1. Yes - previous standalone PCI (NOT as part of staged or hybrid procedure)	21.83
0. No	36.73
2. Yes - as part of a staged or hybrid procedure	36.74
1. Yes - previous standalone PCI (NOT as part of staged or hybrid procedure)	27.7
2. Yes - as part of a staged or hybrid procedure	25.37
0. No	18.43
1. Yes - previous standalone PCI (NOT as part of staged or hybrid procedure)	27.8
0. No	27.93
1. Yes - previous standalone PCI (NOT as part of staged or hybrid procedure)	34.24
0. No	26.67
1. Yes - previous standalone PCI (NOT as part of staged or hybrid procedure)	34.68
0. No	28.8
0. No	23.74
0. No	31.18
0. No	25.72
0. No	29.71
0. No	25.15
0. No	26.05
0. No	31.28
0. No	24.21
2. Yes - as part of a staged or hybrid procedure	25.74

LV function
1. Good (LVEF greater than or equal to 50%)
2. Fair (LVEF 30 - 49%)
1. Good (LVEF greater than or equal to 50%)
3. Poor (LVEF less than 30%)
2. Fair (LVEF 30 - 49%)
1. Good (LVEF greater than or equal to 50%)
1. Good (LVEF greater than or equal to 50%)
1. Good (LVEF greater than or equal to 50%)
1. Good (LVEF greater than or equal to 50%)
1. Good (LVEF greater than or equal to 50%)
1. Good (LVEF greater than or equal to 50%)
1. Good (LVEF greater than or equal to 50%)
1. Good (LVEF greater than or equal to 50%)
1. Good (LVEF greater than or equal to 50%)
1. Good (LVEF greater than or equal to 50%)
1. Good (LVEF greater than or equal to 50%)
2. Fair (LVEF 30 - 49%)
1. Good (LVEF greater than or equal to 50%)
2. Fair (LVEF 30 - 49%)
1. Good (LVEF greater than or equal to 50%)
1. Good (LVEF greater than or equal to 50%)
1. Good (LVEF greater than or equal to 50%)
1. Good (LVEF greater than or equal to 50%)
2. Fair (LVEF 30 - 49%)
1. Good (LVEF greater than or equal to 50%)
1. Good (LVEF greater than or equal to 50%)
1. Good (LVEF greater than or equal to 50%)
2. Fair (LVEF 30 - 49%)
1. Good (LVEF greater than or equal to 50%)
1. Good (LVEF greater than or equal to 50%)
