# Supplementary material for: Cardiac rehabilitation to improve health-related quality of life following trans-catheter aortic valve implantation: a randomised controlled feasibility study: RECOVER–TAVI Pilot, ORCA 4, For the Optimal Restoration of Cardiac Activity Group
Source: Pilot Feasibility Stud. 2018 Dec 13;4:185. doi: 10.1186/s40814-018-0363-8 (PMC6293531; doi:10.1186/s40814-018-0363-8)
Supplement: Supplementary file 4 — Patient Outcomes. (PDF 228 kb) [file 40814_2018_363_MOESM4_ESM.pdf]

					HANDGRIP			
**STUDY No**	**HPAQ_job**	**HPAQ_min**	**HPAQ_TEE_Wk**	**HPAQ_TEE_Day**	**Position**	**Rt(0)/Lt(1)**	**Avrg**	**4MG-1**
**RT-001**	1	30	105	15	2	0	23.33	4.31
**RT-002**								
**RT-003**								
**RT-004**	1	560	1890	270	3	0	30.33	5
**RT-005**	1	340	1160	165.71	3	0	20	5.72
**RT-006**	1	60	210	30	2	0	12.66	5.81
**RT-007**								
**RT-008**	1	90	315	45	3	0	18.66	5.28
**RT-009**								
**RT-010**	1	250	875	125	3	0	43.66	4.68
**RT-011**	1	500	1660	237.14	2	0	13.33	5.88
**RT-012**	1	80	250	35.71	3	0	29	4.53
**RT-013**	1	438	1537.5	219.64	2	0	22.33	4.69
**RT-014**	1	300	1050	150	3	0	29.66	4.19
**RT-015**	1	270	945	135	3	1	20.66	4.18
**RT-016**	1	450	1515	216.42	2	0	15.33	3.84
**RT-017**	1	457.5	1601.25	228.75	3	0	23.06	7.68
**RT-018**	1	10	35	5	2	0	9.33	7.41
**RT-019**	1	55	182.5	26.07	2	0	21	6.41
**RT-020**	1	390	1365	195	2	0	14.56	5.28
**RT-021**	1	220	770	110	3	0	10	4.56
**RT-022**	1	270	900	128.57	3	0	20.66	4.41
**RT-023**	1	140	506.5	72.35	3	0	25.33	4.75
**RT-024**	1	90	315	45	3	0	25	4.81
**RT-025**								
**RT-026**	1	100	350	50	3	1	31.33	3.66
**RT-027**	1	120	375	53.57	3	0	15.33	4.75
**RT-028**	1	70	245	35	2	0	14	4.1

**RT-029**	1	160	612.5	87.5	2	0	28.66	6.75
**RT-030**	1	45	157.5	22.5	3	0	14.66	4.72
**RT-031**								
**RT-032**	1	278.75	891.25	127.32	3	0	21.33	4.88

4MG-2	4MG- fastest	Aids (0 = none, 1 =	CES-D (a)	CES-D(b)	Fried	Nottingham Ext ADL	Edmonton Frail Score
4.35	4.31	0	0	0	1	17	4
4.88	4.88	1	1	1	0	14	4
999	5.72	0	1	2	3	13	8
5.28	5.28	0	0	0	3	20	4
4.63	4.63	0	0	0	0	15	5
4.56	4.56	0	0	0	0	14	4
4.87	4.87	0	0	0	1	19	5
4.03	4.03	0	1	2	3	16	7
4.78	4.69	0	0	0	0	22	4
3.65	3.65	0	0	0	1	16	2
4.28	4.18	0	0	0	1	12	5
3.66	3.66	0	0	0	2	18	7
6.63	6.63	0	1	1	2	14	7
6.81	6.81	1	0	0	3	17	9
5.75	5.75	0	1	0	2	19	7
7.28	5.28	0	0	0	2	13	5
4.53	4.53	0	0	1	1	21	3
4.37	4.37	0	1	2	2	19	4
4.54	4.54	0	0	0	1	18	4
4.91	4.81	0	3	3	3	18	7
3.94	3.66	0	0	0	1	21	3
4.72	4.72	0	0	0	1	15	
4.13	4.1	0	0	0	2	3	2

5.56	5.56	1	1	0	2	11	7
4.37	4.37	0	0	0	2	12	7
4.62	4.62	0	0	0	1	17	4

HADS-Anxiety	HADS-Depression	BP rest	HR-b/l	HR-end	Dysp-b/l	Dysp-end	Fatigue-b/l	fatigue-End
1	1	170/67	70	77	0	1	0	1
2	4							
4	3							
2	2							
4	2							
1	1							
2	2							
7	8							
7	0		72	89	0	2	0	0.5
0	4	137/83	77	89	0	1	0	2
0	5							
3	4							
3	8							
8	3							
1	1							
1	0							
3	2	134/80	72	86	0	3	0.5	3
3	7	148/67	86	104	1	4	0.5	4
0	0	130/58	60	84	0	0	0	1
7	7							
4	4	139/72	74	88	0	0.5	0	0.5
0	0							
3	3							

9	8							
1	4							
1	1	147/87	51	73	0	1	0	0

Distance	Stop/pause (Y=1,N=0)	Reason	Symtoms at end	Oxygen (Y=1, N=0)	Aids (Y=1, N=0)
333	0		0	0	0
327	0		0	0	0
350	0		0	0	0
325	0		0	0	0
330	0		0	0	0
288	0				
430	0		0	0	0

372	0		0	0	0
