# Supplementary material for: Cardiac rehabilitation to improve health-related quality of life following trans-catheter aortic valve implantation: a randomised controlled feasibility study: RECOVER–TAVI Pilot, ORCA 4, For the Optimal Restoration of Cardiac Activity Group
Source: Pilot Feasibility Stud. 2018 Dec 13;4:185. doi: 10.1186/s40814-018-0363-8 (PMC6293531; doi:10.1186/s40814-018-0363-8)
Supplement: Supplementary file 5 — Patient HRQoL Outcomes. (PDF 319 kb) [file 40814_2018_363_MOESM5_ESM.pdf]

Study No	**Gender**	**Age**	**Diabetes**	**Smoking status**	**Creatinine (mmol/l)**
1	female	69	2. Diabetes (oral medicine)	1. Ex smoker	75
2	male	77	0. Not diabetic	0. Never smoked	84
3	male	62	2. Diabetes (oral medicine)	0. Never smoked	67
4	male	79	3. Diabetes (insulin)	0. Never smoked	85
5	male	86	0. Not diabetic	0. Never smoked	145
6	female	74	0. Not diabetic	1. Ex smoker	95
7	female	89	0. Not diabetic	0. Never smoked	79
8	female	86	0. Not diabetic	1. Ex smoker	63
9	female	84	0. Not diabetic	0. Never smoked	68
10	male	90	0. Not diabetic	1. Ex smoker	100
11	female	83	0. Not diabetic	0. Never smoked	77
12	female	85	0. Not diabetic	0. Never smoked	68
13	male	75	0. Not diabetic	1. Ex smoker	179
14	female	87	0. Not diabetic	1. Ex smoker	91
15	male	67	1. Diabetes (dietary control)	0. Never smoked	69
16	male	80	0. Not diabetic	1. Ex smoker	84
17	female	87	0. Not diabetic	0. Never smoked	56
18	male	88	1. Diabetes (dietary control)	0. Never smoked	186
19	female	80	0. Not diabetic	1. Ex smoker	72
20	male	73	0. Not diabetic	0. Never smoked	125
21	male	86	0. Not diabetic	1. Ex smoker	90
22	female	89	0. Not diabetic	1. Ex smoker	72
23	female	82	1. Diabetes (dietary control)	0. Never smoked	58
24	female	77	0. Not diabetic	0. Never smoked	87
25	male	91	0. Not diabetic	1. Ex smoker	118
26	male	83	0. Not diabetic	1. Ex smoker	164
27	male	88	0. Not diabetic	1. Ex smoker	84
28	female	83	0. Not diabetic	0. Never smoked	64
29	female	86	0. Not diabetic	0. Never smoked	69
30	male	81	0. Not diabetic	1. Ex smoker	111
31	female	83	0. Not diabetic	1. Ex smoker	56
32	female	85	0. Not diabetic	0. Never smoked	59
33	male	83	2. Diabetes (oral medicine)	1. Ex smoker	113
35	female	91	0. Not diabetic	0. Never smoked	56
35	female	88	0. Not diabetic	0. Never smoked	67
36	female	65	0. Not diabetic	0. Never smoked	95
37	female	82	0. Not diabetic	0. Never smoked	87
38	male	87	3. Diabetes (insulin)	1. Ex smoker	135

Previous MI and interval be	History of pulmonary diseas
5. MI >90 days	1. COAD/emphysema
5. MI >90 days	1. COAD/emphysema
0. No previous MI	0. No pulmonary disease
5. MI >90 days	2. Asthma
0. No previous MI	0. No pulmonary disease
0. No previous MI	1. COAD/emphysema
0. No previous MI	0. No pulmonary disease
0. No previous MI	2. Asthma
0. No previous MI	0. No pulmonary disease
5. MI >90 days	1. COAD/emphysema
0. No previous MI	0. No pulmonary disease
0. No previous MI	0. No pulmonary disease
0. No previous MI	0. No pulmonary disease
5. MI >90 days	0. No pulmonary disease
0. No previous MI	0. No pulmonary disease
0. No previous MI	1. COAD/emphysema
0. No previous MI	0. No pulmonary disease
0. No previous MI	2. Asthma
5. MI >90 days	0. No pulmonary disease
0. No previous MI	0. No pulmonary disease
0. No previous MI	1. COAD/emphysema
0. No previous MI	2. Asthma
0. No previous MI	0. No pulmonary disease
0. No previous MI	3. Other significant pulmonary disease
5. MI >90 days	0. No pulmonary disease
5. MI >90 days	0. No pulmonary disease
5. MI >90 days	0. No pulmonary disease
0. No previous MI	0. No pulmonary disease
0. No previous MI	0. No pulmonary disease
0. No previous MI	0. No pulmonary disease
0. No previous MI	0. No pulmonary disease
0. No previous MI	0. No pulmonary disease
5. MI >90 days	1. COAD/emphysema
0. No previous MI	0. No pulmonary disease
0. No previous MI	0. No pulmonary disease
0. No previous MI	0. No pulmonary disease
0. No previous MI	0. No pulmonary disease
0. No previous MI	0. No pulmonary disease

Pre-operative heart rhythm	Previous cardiac surgery
0. Sinus rhythm	0. No
0. Sinus rhythm	1. Previous CABG
1. Atrial fibrillation / flutter	0. No
0. Sinus rhythm	0. No
6. Paced rhythm	2. Previous valve operation
0. Sinus rhythm	0. No
0. Sinus rhythm	0. No
1. Atrial fibrillation / flutter	0. No
1. Atrial fibrillation / flutter	0. No
0. Sinus rhythm	0. No
1. Atrial fibrillation / flutter	2. Previous valve operation
6. Paced rhythm	0. No
0. Sinus rhythm	1. Previous CABG
0. Sinus rhythm	0. No
0. Sinus rhythm	1. Previous CABG
1. Atrial fibrillation / flutter,6. Paced rhythm	1. Previous CABG
0. Sinus rhythm	0. No
1. Atrial fibrillation / flutter	0. No
0. Sinus rhythm	0. No
0. Sinus rhythm	1. Previous CABG
0. Sinus rhythm	1. Previous CABG
0. Sinus rhythm	0. No
1. Atrial fibrillation / flutter	0. No
0. Sinus rhythm,1. Atrial fibrillation / flutter	0. No
0. Sinus rhythm	1. Previous CABG
0. Sinus rhythm	1. Previous CABG
0. Sinus rhythm	0. No
0. Sinus rhythm	0. No
6. Paced rhythm	1. Previous CABG
1. Atrial fibrillation / flutter	0. No
1. Atrial fibrillation / flutter	1. Previous CABG
0. Sinus rhythm	0. No
1. Atrial fibrillation / flutter	1. Previous CABG
2. First degree heart block,4. LBBB	0. No
0. Sinus rhythm	0. No
1. Atrial fibrillation / flutter	0. No
2. First degree heart block,4. LBBB	0. No
0. Sinus rhythm	0. No

Previous PCI
0. No
1. Yes - previous standalone PCI (NOT as part of staged or hybrid procedure)
0. No
1. Yes - previous standalone PCI (NOT as part of staged or hybrid procedure)
0. No
0. No
2. Yes - as part of a staged or hybrid procedure
0. No
0. No
2. Yes - as part of a staged or hybrid procedure
0. No
0. No
1. Yes - previous standalone PCI (NOT as part of staged or hybrid procedure)
0. No
0. No
0. No
2. Yes - as part of a staged or hybrid procedure
0. No
1. Yes - previous standalone PCI (NOT as part of staged or hybrid procedure)
0. No
0. No
0. No
0. No
0. No
0. No
0. No
2. Yes - as part of a staged or hybrid procedure
0. No
1. Yes - previous standalone PCI (NOT as part of staged or hybrid procedure)
0. No
0. No
0. No
0. No
0. No
0. No
0. No
0. No
0. No

	Scales	
**LV function**	Physical Limitation	Symptom Stability
1. Good (LVEF greater than or equal to 50%)	100	75
2. Fair (LVEF 30 - 49%)	80	75
1. Good (LVEF greater than or equal to 50%)	58.33333333	50
2. Fair (LVEF 30 - 49%)	85	50
1. Good (LVEF greater than or equal to 50%)	75	50
1. Good (LVEF greater than or equal to 50%)	70	50
1. Good (LVEF greater than or equal to 50%)	100	50
1. Good (LVEF greater than or equal to 50%)	-	50
1. Good (LVEF greater than or equal to 50%)	41.66666667	25
1. Good (LVEF greater than or equal to 50%)	65	50
1. Good (LVEF greater than or equal to 50%)	40	50
1. Good (LVEF greater than or equal to 50%)	41.66666667	50
1. Good (LVEF greater than or equal to 50%)	58.33333333	0
1. Good (LVEF greater than or equal to 50%)	29.16666667	75
1. Good (LVEF greater than or equal to 50%)	100	50
1. Good (LVEF greater than or equal to 50%)	75	50
1. Good (LVEF greater than or equal to 50%)	45	50
1. Good (LVEF greater than or equal to 50%)	95	50
1. Good (LVEF greater than or equal to 50%)	90	50
1. Good (LVEF greater than or equal to 50%)	100	50
1. Good (LVEF greater than or equal to 50%)	41.66666667	25
1. Good (LVEF greater than or equal to 50%)	100	50
1. Good (LVEF greater than or equal to 50%)	100	25
1. Good (LVEF greater than or equal to 50%)	100	50
1. Good (LVEF greater than or equal to 50%)	-	50
2. Fair (LVEF 30 - 49%)	37.5	50
1. Good (LVEF greater than or equal to 50%)	-	50
1. Good (LVEF greater than or equal to 50%)	91.66666667	100
2. Fair (LVEF 30 - 49%)	41.66666667	75
1. Good (LVEF greater than or equal to 50%)	-	50
1. Good (LVEF greater than or equal to 50%)	100	50
1. Good (LVEF greater than or equal to 50%)	93.75	50
2. Fair (LVEF 30 - 49%)	68.75	75
1. Good (LVEF greater than or equal to 50%)	54.16666667	100
1. Good (LVEF greater than or equal to 50%)	75	50
3. Poor (LVEF less than 30%)	91.66666667	75
1. Good (LVEF greater than or equal to 50%)	79.16666667	50
1. Good (LVEF greater than or equal to 50%)	20.83333333	0

Symptom Frequency	Symptom Burden	Total Symptom Score	Self-Efficacy	Quality of Life
70.83333333	66.66666667	68.75	25	91.66666667
70.83333333	66.66666667	68.75	87.5	58.33333333
31.25	58.33333333	44.79166667	50	33.33333333
91.66666667	83.33333333	87.5	100	91.66666667
75	75	75	37.5	75
85.41666667	83.33333333	84.375	87.5	91.66666667
75	75	75	100	91.66666667
100	100	100	100	100
4.166666667	0	2.083333333	87.5	16.66666667
72.91666667	100	86.45833333	62.5	100
37.5	66.66666667	52.08333333	100	33.33333333
54.16666667	50	52.08333333	100	58.33333333
58.33333333	41.66666667	50	87.5	25
62.5	83.33333333	72.91666667	62.5	75
100	100	100	87.5	100
50	33.33333333	41.66666667	100	33.33333333
54.16666667	58.33333333	56.25	75	16.66666667
100	100	100	100	100
91.66666667	100	95.83333333	100	91.66666667
83.33333333	91.66666667	87.5	100	91.66666667
79.16666667	75	77.08333333	75	58.33333333
95.83333333	100	97.91666667	100	100
45.83333333	50	47.91666667	100	91.66666667
100	100	100	100	100
70.83333333	83.33333333	77.08333333	100	100
91.66666667	75	83.33333333	100	75
100	100	100	100	75
100	100	100	87.5	91.66666667
70.83333333	66.66666667	68.75	87.5	50
66.66666667	75	70.83333333	100	75
83.33333333	83.33333333	83.33333333	100	75
79.16666667	100	89.58333333	62.5	83.33333333
58.33333333	83.33333333	70.83333333	37.5	66.66666667
72.91666667	66.66666667	69.79166667	37.5	66.66666667
100	100	100	87.5	91.66666667
91.66666667	83.33333333	87.5	75	58.33333333
58.33333333	66.66666667	62.5	87.5	91.66666667
8.333333333	16.66666667	12.5	25	16.66666667

			Raw Responses									
Social Limitation	Overall Summary	Clinical Summary	1A	1B	1C	1D	1E	1F	2	3	4	5
100	90.10416667	84.375	5	5	5	6	6	6	4	1	2	6
58.33333333	66.35416667	74.375	5	5	4	2	5	6	4	5	6	3
43.75	45.05208333	51.5625	1	2	4	4	4	5	3	2	4	3
100	91.04166667	86.25	5	5	5	5	2	6	3	5	6	5
-	75	75	4	4	4	4	6	6	3	5	6	1
100	86.51041667	77.1875	5	5	4	4	6	1	3	4	4	7
-	88.88888889	87.5	5	5	5	6	6	6	3	4	4	4
-	100	100	5	5	6	6	6	6	3	5	6	7
25	21.35416667	21.875	4	4	2	1	4	1	2	1	1	2
37.5	72.23958333	75.72916667	5	5	3	1	4	6	3	2	5	5
41.66666667	41.77083333	46.04166667	3	4	2	1	3	6	3	5	6	2
58.33333333	52.60416667	46.875	4	3	3	2	3	1	3	5	6	1
31.25	41.14583333	54.16666667	4	5	4	2	4	1	1	5	6	2
58.33333333	58.85416667	51.04166667	3	3	3	2	1	1	4	3	4	4
100	100	100	5	5	5	5	5	5	6	5	5	7
-	50	58.33333333	6	5	5	6	2	6	3	3	3	3
33.33333333	37.8125	50.625	5	5	1	2	1	6	3	1	3	4
100	98.75	97.5	5	5	5	4	5	6	6	5	6	7
75	88.125	92.91666667	5	5	4	4	5	6	3	5	6	6
100	94.79166667	93.75	5	5	5	5	6	6	3	5	6	3
62.5	59.89583333	59.375	3	3	2	3	2	3	2	5	6	5
-	99.30555556	98.95833333	5	5	5	6	5	6	6	5	6	6
100	84.89583333	73.95833333	5	5	5	5	6	6	2	3	3	2
100	100	100	5	5	5	6	6	6	3	5	5	7
-	88.54166667	77.08333333	2	6	1	6	6	6	3	1	3	6
41.66666667	59.375	60.41666667	3	3	3	3	2	1	3	5	5	5
-	87.5	100	4	6	5	6	6	6	3	5	6	7
93.75	94.27083333	95.83333333	5	5	5	4	5	4	5	5	6	7
75	58.85416667	55.20833333	4	3	3	2	2	2	4	5	6	4
-	72.91666667	70.83333333	6	6	6	6	6	6	3	1	3	6
100	89.58333333	91.66666667	5	5	5	6	5	6	3	5	5	3
58.33333333	81.25	91.66666667	5	5	5	4	6	6	3	5	6	2
25	57.8125	69.79166667	5	5	3	2	6	6	4	1	5	7
66.66666667	64.32291667	61.97916667	5	4	3	3	3	1	5	4	4	4
81.25	86.97916667	87.5	5	5	4	4	5	1	6	5	6	7
75	78.125	89.58333333	5	5	5	4	5	4	4	5	6	6
83.33333333	79.16666667	70.83333333	5	5	5	5	4	1	3	1	4	4
16.66666667	16.66666667	16.66666667	3	3	1	2	1	1	1	1	1	2

6	7	8	9	10	11	12	13	14	15A	15B	15C	15D
4	7	6	5	2		5	4	5	5	6	6	5
3	4	3	5	4	5	2	4	4	3	3	4	6
4	2	2	3	2	4	2	2	3	2	2	5	2
3	7	6	5	5	5	5	4	5	6	6	5	5
2	7	6	5	3	2	5	5	2	6	6	6	6
5	5	4	5	4	5	4	5	5	5	5	5	6
4	7	4	4	5	5	4	5	5	6	6	3	6
6	7	6	5	5		5	5	5	6	6	5	6
1	1	1	1	4	5	1	1	3	1	4	2	1
5	7	5	5	4	3	5	5	5	3	6	2	6
4	3	2	1	5		2	3	2	1	2	5	6
2	2	2	5	5	5	3	2	5	3	2	5	6
1	2	2	5	5	4	3	1	2	2	2	3	2
4	4	5	5	4	3	5	3	4	3	3	4	6
6	7	5	5	5	4	5	5	5	5	5	5	5
2	2	2	5	5		2	2	3	3	6	6	6
3	5	4	5	5	3	2	1	2	2	2	3	6
6	7	6	5	5		5	5	5	5	5	5	
5	6	5	5	5	5	4	5	5	3	4	5	6
4	7	6	5	5		5	4	5	5	5	5	5
4	4	3	5	4		3	3	4	2	4	3	5
5	7	6	5	5	5	5	5	5	6	5	6	6
2	2	4	5	5	5	5	5	4	5	5	5	6
6	7	5	5	5	5	5	5	5	6	6	5	5
5	7	6	5	5		5	5	5	6	4	6	6
2	7	6	5	5	5	2	5	5	3	4	1	6
5	7	6	5	5		5	4	3	6	6	5	6
5	7	5	5	4	5	5	4	5	5	4	5	5
3	3	3	5	4	5	3	3	3	6	4	4	6
4	6	5	5	5		5	4	3	6	6	6	6
3	7	5	5	5		5	2	5	5	6	6	5
5	7	5	5	5	2	5	5	3	3	4	3	6
5	3	3	5	4	1	3	3	5	2	2	6	6
3	5	4	5	1	4	4	4	3	3	3	5	6
5	7	5	5	5	4	5	4	5	2	5	5	5
4	6	4	5	4	4	4	3	3	3	4	5	4
3	6	4	5	5	4	5	4	5	4	4	5	6
2	2	2	1	2	2	2	1	2	2	2	1	6
